# Regulation of ER Stress‐Associated Gene Expression by Lifeguard in Human Breast Cancer Cells

**DOI:** 10.1002/cnr2.70687

**Published:** 2026-10-01

**Authors:** Inga Nebel, Sarah Strauß, Frederik Schlottmann, Lena Maria Herrmann, Peter M. Vogt, Vesna Bucan

**Affiliations:** ^1^ Department of Plastic, Aesthetic, Hand and Reconstructive Surgery Hannover Medical School Hannover Germany

**Keywords:** apoptosis, endoplasmic reticulum, gene expression, Lifeguard

## Abstract

**Background:**

Despite significant advances in breast cancer therapy, resistance to apoptosis remains a major obstacle to successful treatment. Lifeguard (LFG), an antiapoptotic membrane protein, has been implicated in breast cancer progression; however, its role in endoplasmic reticulum (ER) stress‐associated molecular responses and apoptosis‐related gene regulation remains incompletely understood.

**Aims:**

This study aimed to investigate molecular changes associated with LFG‐targeted siRNA treatment and reduced LFG mRNA expression in human breast cancer cells.

**Methods and Results:**

The triple‐negative breast cancer cell line MDA‐MB‐231 and the non‐tumorigenic mammary epithelial cell line MCF10A were transfected with LFG‐specific siRNA. LFG mRNA expression was analyzed by quantitative RT‐PCR. Furthermore, ER stress‐ and apoptosis‐related gene expression was examined using an RT^2^ Profiler PCR Array, and intracellular calcium‐associated fluorescence was assessed by fluorescence‐based calcium imaging. LFG mRNA expression was markedly reduced following LFG‐specific siRNA treatment in MDA‐MB‐231 cells, whereas a more moderate reduction was observed in MCF10A cells. LFG‐targeted siRNA treatment was associated with altered intracellular calcium‐associated fluorescence and differential expression of several ER stress‐ and apoptosis‐related genes, including upregulation of CREB3L3, SREBF1, and INHBE, as well as downregulation of EDEM1, HSPA2, and RRM2.

**Conclusions:**

These findings demonstrate that LFG‐targeted siRNA treatment and reduced LFG mRNA expression are associated with changes in intracellular calcium‐related responses and ER stress‐ and apoptosis‐associated gene expression, particularly in MDA‐MB‐231 cells. Further studies are required to determine the underlying mechanisms and establish the functional role of LFG in these cellular responses.

## 1 Introduction

Breast cancer remains the most frequently diagnosed malignancy among women worldwide and one of the leading causes of cancer‐related mortality among women [1, 2]. Despite substantial advances in early detection and treatment, including targeted therapies, chemotherapy, and surgical techniques, drug resistance remains a major clinical challenge, frequently resulting in tumor recurrence and metastasis [3]. Recent studies have suggested that one of the mechanisms by which cancer cells evade apoptosis and thereby chemotherapy‐induced cell death is the overexpression of the antiapoptotic protein Lifeguard (LFG). LFG has been proposed to inhibit apoptosis, thereby promoting cancer cell survival and resistance to chemotherapeutic agents [4]. Consequently, modulation of LFG expression has been proposed as a potential strategy to sensitize cancer cells to apoptosis and thereby overcome therapy resistance [5, 6].

The LFG protein, also known as Fas Apoptotic Inhibitory Molecule 2 (FAIM2), belongs to a family of evolutionarily conserved transmembrane proteins involved in the regulation of apoptosis [7, 8]. This protein has been implicated in the regulation of apoptotic pathways by modulating cell death responses, particularly in response to extrinsic signals. It is predominantly localized to cellular membranes, with notable concentrations in the endoplasmic reticulum (ER) and Golgi apparatus [8]. This subcellular localization suggests that LFG may contribute to the regulation of cellular responses to ER stress, although the underlying molecular mechanisms remain incompletely understood.

Previous studies, including work from our group, demonstrated that LFG is overexpressed in human breast cancer tissues and cell lines, where it promotes cell survival by inhibiting Fas‐mediated apoptosis [7, 9]. Increased LFG expression has also been associated with enhanced tumor cell survival, tumorigenesis, and metastatic potential [4, 7, 9].

Interestingly, the effects of LFG on apoptosis and its potential involvement in tumor malignancy extend beyond breast cancer. Recent studies have reported upregulation of LFG (FAIM2) expression in other malignancies, including colorectal cancer, where elevated FAIM2 expression has been associated with tumor development and poor clinical outcome [10, 11]. In addition, LFG overexpression has been shown to promote cell growth and bone metastasis in patients with non‐small cell lung cancer (NSCLC), further illustrating the role of LFG as a key modulator of cancer cell viability and metastasis [12].

Although the antiapoptotic function of LFG in Fas receptor‐mediated apoptosis has been well established [6, 8], considerably less is known about its role in ER stress‐associated molecular responses. Given its predominant localization to the ER, LFG may contribute to the regulation of ER stress‐related pathways involved in cellular homeostasis and apoptosis [8]. Therefore, the aim of the present study was to investigate the effects of small interfering RNA (siRNA)‐mediated LFG silencing on ER stress‐associated gene expression and intracellular calcium homeostasis using the triple‐negative breast cancer cell line MDA‐MB‐231 and the non‐tumorigenic mammary epithelial cell line MCF10A.

## 2 Methods

### 2.1 LFG Protein Structure

A three‐dimensional (3D) model of the human LFG protein was generated using the automated SWISS‐MODEL server (Basel, Switzerland) as previously described [12]. The amino acid sequence of human LFG was obtained from the UniProt database and used as input for homology modeling. The quality of the predicted model was assessed using the Global Model Quality Estimation (GMQE) score provided by SWISS‐MODEL. The structural model was included solely to illustrate the predicted membrane topology and domain organization of LFG and was not used for functional or structure–function analyses.

### 2.2 Cell Lines and Culture Conditions

The human breast cancer cell line MDA‐MB‐231 (MDA) was obtained from the American Type Culture Collection (ATCC, Rockville, MD, USA) and cultured in Dulbecco's modified Eagle's medium (DMEM; PAA, Cölbe, Germany) supplemented with 10% fetal calf serum (FCS; Merck KGaA, Darmstadt, Germany). The non‐tumorigenic human mammary epithelial cell line MCF10A was obtained from ATCC and cultured in DMEM/F12 medium (Gibco, Thermo Fisher Scientific, Waltham, MA, USA) supplemented with 5% horse serum (Sigma‐Aldrich, St. Louis, MO, USA), 100 ng/mL cholera toxin, 20 ng/mL epidermal growth factor, 0.01 mg/mL insulin, and 500 ng/mL hydrocortisone.

Cells were routinely maintained at 37°C in a humidified incubator containing 5% CO_2_. Cells were subcultured three times per week using 0.25% trypsin/0.53 mM ethylenediaminetetraacetic acid (EDTA).

### 2.3 siRNA Transfection

MDA‐MB‐231 and MCF10A cells were transfected with a siRNA targeting LFG (LFG‐650: 5′‐CCTCCTACCCTTCCAATATGT‐3′; Sirion Biotech, Munich, Germany) or the corresponding non‐targeting control siRNA (Sirion Biotech, Munich, Germany). Cells were seeded at a density of 2 × 10^4^ cells/cm^2^ and cultured under standard conditions (37°C in a humidified atmosphere containing 5% CO₂). For transfection, siRNA was diluted to a final concentration of 60 nM in 200 μL of culture medium and mixed with 6 μL Lipofectamine 2000 (Thermo Fisher Scientific, Waltham, MA, USA) according to the manufacturer's instructions. After incubation for 5 min at room temperature, the transfection mixture was added to 25 cm^2^ culture flasks. Cells were incubated for 24 or 48 h before subsequent analyses. The corresponding non‐targeting siRNA served as the negative transfection control throughout all experiments.

### 2.4 RT^2^ Profiler PCR Array

To investigate the effects of LFG silencing on ER stress‐associated gene expression, total RNA was isolated from MDA‐MB‐231 and MCF10A cells following siRNA transfection and analyzed using the RT^2^ Profiler PCR Array System (Qiagen, Venlo, The Netherlands) according to the manufacturer's instructions. PCR amplification was performed using SsoFast EvaGreen Supermix (Bio‐Rad, Watford, UK) with an initial denaturation at 95°C for 10 min, followed by 40 cycles of 95°C for 15 s, 55°C for 40 s, and 72°C for 30 s (iCycler, Bio‐Rad, Watford, UK). Reactions were carried out in a final volume of 15 μL. Raw *C*_t_ values were analyzed using the Qiagen GeneGlobe Data Analysis Center according to the manufacturer's recommendations. Gene expression levels were normalized to the reference gene ACTB (β‐actin) included in the RT^2^ Profiler PCR Array. Relative gene expression was calculated using the 2^−ΔΔCt^ method, using the corresponding non‐targeting control siRNA‐transfected cells as the calibrator. Genes with *C*_t_ values ≥ 35 were considered below the detection limit according to the manufacturer's recommendations. Only genes fulfilling both the predefined fold‐change threshold (≥ 2 or ≤ −2) and the manufacturer's quality control criteria were considered differentially expressed.

### 2.5 Ca^2+^ Assay

To assess intracellular calcium mobilization following LFG silencing, MDA‐MB‐231 and MCF10A cells were seeded at a density of 1 × 10^4^ cells per well in 96‐well plates and incubated for 24 h under standard culture conditions. Cells were then transfected with 60 nM LFG‐specific siRNA or the corresponding non‐targeting control siRNA and incubated for 48 h. Subsequently, cells were stimulated with 100 ng/mL agonistic anti‐Fas antibody (clone CH11; Abcam, Cambridge, UK) for an additional 24 h. Intracellular calcium mobilization was assessed using the ScreenQuest Calbryte‐520 Calcium Assay Kit (AAT Bioquest) according to the manufacturer's instructions. Briefly, 100 μL of Calbryte‐520 dye loading solution was added to each well and incubated for 60 min at 37°C. Fluorescence images were acquired using a fluorescence microscope equipped with FITC filter settings (excitation 492 nm, emission 521 nm) under identical exposure conditions for all experimental groups.

### 2.6 Statistical Analysis

Unless otherwise stated, experiments were performed in three independent biological replicates. Data are presented as mean ± standard deviation (SD). Statistical comparisons between LFG‐specific siRNA‐treated cells and the corresponding non‐targeting control siRNA‐treated cells were performed using a two‐tailed Student's *t*‐test. A *p*‐value < 0.05 was considered statistically significant.

## 3 Results

### 3.1 LFG Protein Structure

The predicted 3D structure of the human Lifeguard (LFG/FAIM2) protein was generated using the SWISS‐MODEL server and is shown in Figure 1. The model revealed a protein comprising seven transmembrane domains, consistent with the reported membrane localization of LFG (Figure 1A). The quality of the predicted structure was assessed using the local quality estimate and the QMEAN *Z*‐score provided by SWISS‐MODEL (Figure 1B,C). While minor regions of lower confidence were observed in the local quality assessment, the overall model quality was within the expected range for homology‐based structural models. The predicted structure was included to provide structural context for LFG and its membrane topology. No functional or structure–function analyses were performed based on this model.

**FIGURE 1:**
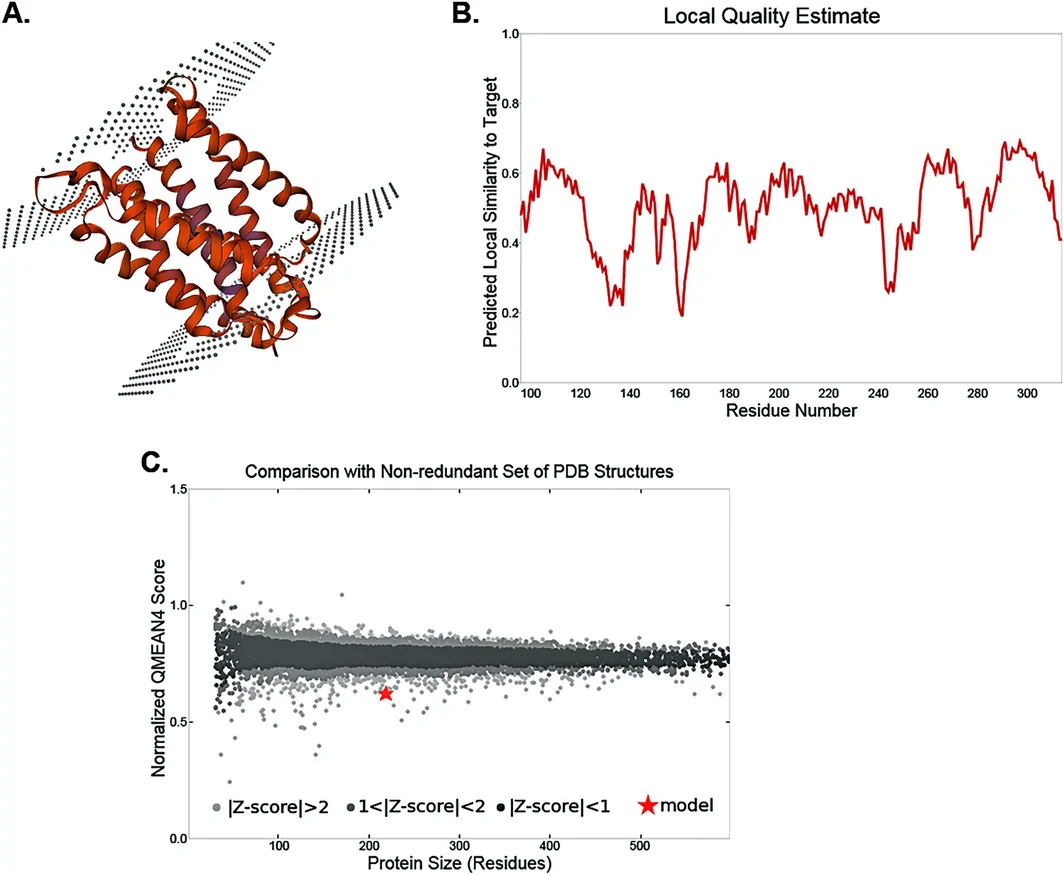
Predicted three‐dimensional structure of the human Lifeguard (LFG/FAIM2) protein generated using the SWISS‐MODEL server. (A) Predicted three‐dimensional model of the human LFG protein illustrating its seven transmembrane domains and membrane‐associated topology. (B) Local quality assessment of the predicted LFG structure generated by SWISS‐MODEL. Higher local quality scores indicate greater confidence in the predicted structure, whereas lower scores indicate regions of reduced prediction reliability. (C) Global quality assessment of the predicted LFG model based on the QMEAN *Z*‐score provided by SWISS‐MODEL. The red star indicates the predicted LFG model relative to experimentally resolved protein structures deposited in the Protein Data Bank (PDB). The structural model is presented to illustrate the predicted membrane topology of LFG and was not used for functional or structure–function analyses.

### 3.2 Expression of LFG in MDA‐MB‐231 and MCF10A Cells Following siRNA‐Mediated Silencing

To investigate the effects of LFG‐specific siRNA, MDA‐MB‐231 and non‐tumorigenic MCF10A cells were transfected and analyzed after 24 and 48 h. LFG mRNA expression was evaluated by quantitative RT‐PCR (Figure 2A). Quantitative RT‐PCR showed that untreated MDA‐MB‐231 cells exhibited higher basal LFG mRNA expression than untreated MCF10A cells (Figure 2A). In MDA‐MB‐231 cells, LFG mRNA expression decreased after 24 h and was strongly reduced after 48 h of LFG‐specific siRNA treatment. In MCF10A cells, which displayed lower basal LFG mRNA expression, a more moderate reduction was observed following LFG‐specific siRNA treatment.

**FIGURE 2:**
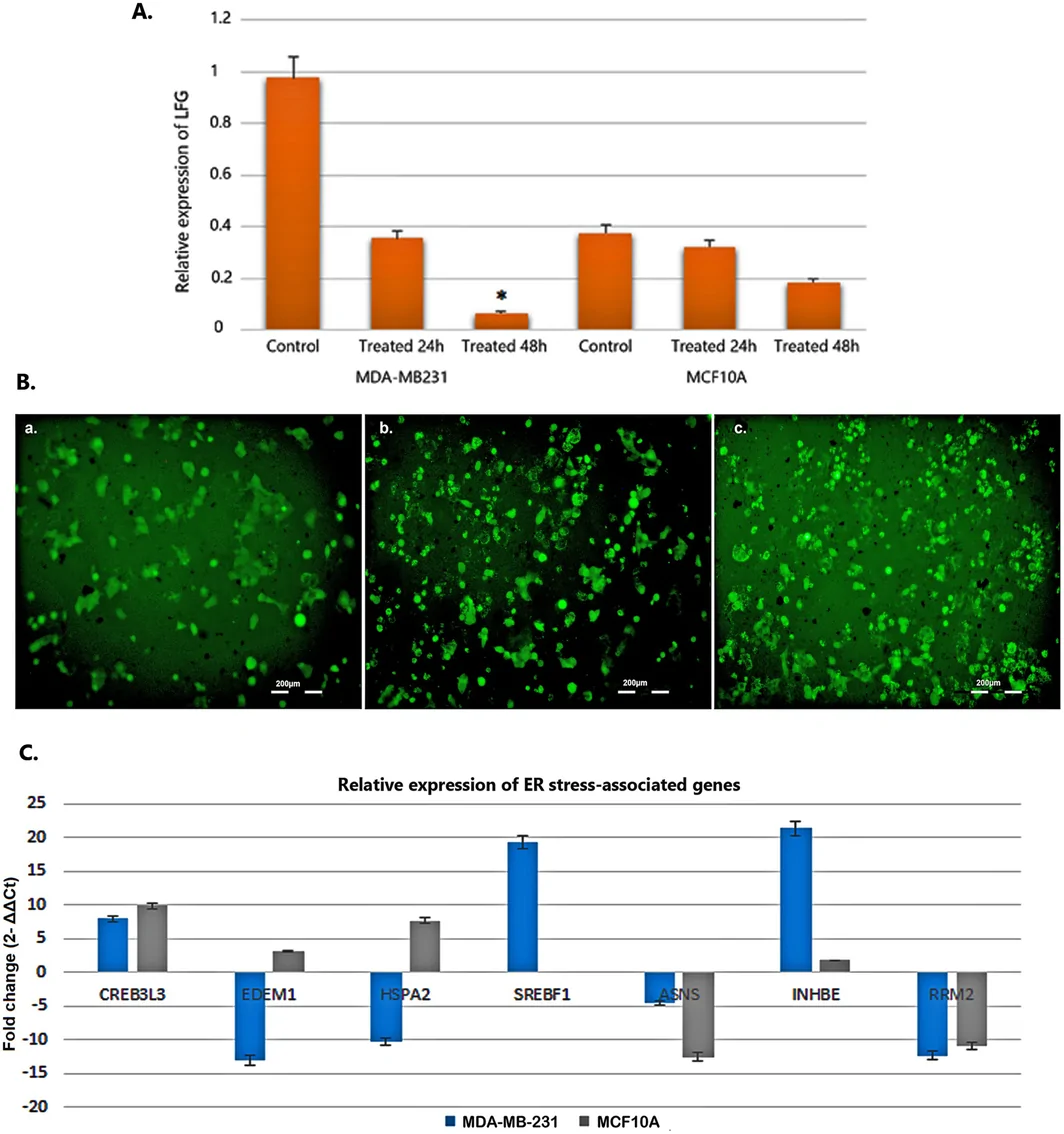
Molecular changes associated with LFG‐specific siRNA treatment in MDA‐MB‐231 and MCF10A cells. (A) LFG mRNA expression measured by qRT‐PCR after 24 and 48 h. Expression was normalized to ACTB and calculated using the 2^−ΔΔCt^ method with MDA‐MB‐231 control cells as the calibrator. Data represent mean ± SD from three independent experiments. *p < 0.05 versus the corresponding control (Student's *t*‐test). (B) Representative fluorescence images showing intracellular Ca^2+^‐associated fluorescence in MDA‐MB‐231 cells following LFG‐specific siRNA transfection. Images shown are representative of three independent biological experiments (*n* = 3). Cells were imaged using identical microscope settings, exposure time, gain, and illumination intensity for all experimental groups. Green fluorescence represents intracellular calcium‐associated fluorescence. Scale bar = 200 μm. (C) Relative expression of selected ER stress‐ and apoptosis‐related genes in MDA‐MB‐231 and MCF10A cells 48 h after LFG‐specific siRNA transfection, determined using the RT^2^ Profiler PCR Array. Gene expression was normalized to ACTB (β‐actin) and calculated using the 2^−ΔΔCt^ method with the corresponding non‐targeting siRNA‐transfected cells as the calibrator. Genes with a fold change ≥ 2 or ≤ −2 were considered differentially expressed. Data are presented as mean ± SD from three independent biological experiments (*n* = 3). Statistical comparisons were performed using an unpaired two‐tailed Student's *t*‐test, with *p* < 0.05 considered statistically significant. Error bars represent standard deviation (SD). The asterisk indicates statistical significance compared with the control group (*p* < 0.05)

### 3.3 Impact of LFG Silencing on Intracellular Calcium Homeostasis and ER Stress‐Associated Gene Expression

Figure 2B shows representative fluorescence images of MDA‐MB‐231 cells following LFG‐specific siRNA treatment. Compared with untreated control cells, LFG‐specific siRNA‐treated cells exhibited a progressive reduction in calcium‐associated fluorescence after 24 and 48 h, indicating changes in intracellular calcium‐associated fluorescence under the experimental conditions.

ER stress‐associated gene expression following LFG‐specific siRNA treatment after 48 h is shown in Figure 2C. Differential expression of several genes involved in ER stress‐associated cellular responses was observed. In MDA‐MB‐231 cells, CREB3L3, SREBF1, and INHBE were upregulated, whereas EDEM1, HSPA2, and RRM2 were downregulated. In MCF10A cells, expression changes were generally less pronounced, although ASNS and RRM2 were reduced following LFG‐specific siRNA treatment.

Overall, these findings demonstrate that LFG‐specific siRNA treatment and the associated reduction in LFG mRNA expression were accompanied by distinct changes in intracellular calcium‐associated fluorescence and ER stress‐ and apoptosis‐related gene expression. The differentially expressed genes identified by the RT^2^ Profiler PCR Array are summarized in Table 1 according to their reported biological functions.

**TABLE 1:** Differentially expressed ER stress‐associated genes identified in MDA‐MB‐231 cells following LFG‐specific siRNA transfection (48 h).

Gene	Regulation in MDA‐MB‐231 cells	Functional category
CREB3L3	Upregulated	Transcription factors
EDEM1	Downregulated	Endoplasmic reticulum protein folding quality control
HSPA2	Downregulated	Unfolded protein binding
SREBF1	Upregulated	Protein disulfide isomerization
ASNS	Downregulated	Pathway activity signature genes
INHBE	Upregulated	Pathway activity signature genes
PRM2	Downregulated	Pathway activity signature genes

*Note:* Genes exhibiting a fold change ≥ 2 or ≤ −2 relative to non‐targeting siRNA‐transfected MDA‐MB‐231 cells were considered differentially expressed. Functional categories are based on the annotation provided in the RT^2^ Profiler PCR Array (Qiagen).

## 4 Discussion

The present study provides further insight into the potential role of Lifeguard (LFG/FAIM2) in breast cancer cells, particularly in the triple‐negative breast cancer cell line MDA‐MB‐231. A particular strength of the study is the integration of complementary approaches, including structural modeling, time‐dependent LFG mRNA expression analysis, measurements of intracellular calcium‐sensitive fluorescence, and the analysis of selected genes associated with ER stress, metabolism, and apoptosis. Moreover, the direct comparison between MDA‐MB‐231 cells and non‐tumorigenic MCF10A mammary epithelial cells enabled the identification of cell type‐dependent molecular response patterns. These findings extend previous studies describing the expression and antiapoptotic activity of LFG in breast cancer cells [3, 4, 6, 9].

Structural prediction suggested that LFG adopts a heptahelical transmembrane topology, consistent with its classification as a member of the membrane‐associated LFG/TMBIM protein family [7]. The QMEAN *Z*‐score and local quality estimates generated using SWISS‐MODEL supported the overall plausibility of the predicted model, although regions with lower local model quality were identified [12]. The model should therefore be considered a computational prediction rather than experimental validation of LFG structure. Nevertheless, the predicted membrane topology is consistent with the classification of LFG as a membrane‐associated inhibitor of death receptor‐mediated apoptosis [7, 8].

At the transcript level, untreated MDA‐MB‐231 cells exhibited higher basal LFG mRNA expression than MCF10A cells. Transfection with LFG‐specific siRNA produced a time‐dependent reduction in LFG mRNA expression in MDA‐MB‐231 cells, with the strongest effect observed after 48 h. A more moderate response was detected in MCF10A cells. These findings demonstrate a cell type‐dependent transcriptional response to LFG‐specific siRNA treatment, with a more pronounced reduction in LFG mRNA expression in MDA‐MB‐231 cells than in non‐tumorigenic MCF10A cells.

Treatment with LFG‐specific siRNA was also associated with reduced intracellular calcium‐sensitive fluorescence. This observation is consistent with a potential relationship between the cellular response to LFG‐specific siRNA treatment and intracellular calcium regulation. LFG has previously been reported to inhibit Fas ligand‐induced ER calcium release, thereby interfering with an important step in apoptosis induction [5]. More generally, ER calcium signaling plays a central role in the regulation of cellular homeostasis, ER stress, and apoptosis [13, 14]. In addition, Pan et al. demonstrated that increased FAIM2 transcription was associated with dedifferentiation and enhanced viability of osteosarcoma cells [15]. Together, these findings support further investigation of the relationship between LFG expression, LFG‐targeted siRNA treatment, cellular stress responses, calcium regulation, and tumor cell survival.

Gene expression analysis revealed increased expression of CREB3L3, SREBF1, and INHBE in MDA‐MB‐231 cells, whereas EDEM1 and HSPA2 expression was reduced. These results are consistent with a molecular response involving genes associated with ER stress, lipid metabolism, protein homeostasis, and cellular survival. FAIM2‐associated regulatory networks have also been described in hepatocellular carcinoma and NSCLC [11, 16, 17]. In addition, the NFKB1‐miR‐612‐FAIM2 regulatory pathway has been implicated in neurofibromatosis type 1 [18], illustrating that FAIM2 can be regulated through different transcriptional and noncoding RNA‐dependent mechanisms in distinct cellular contexts.

The reduction in RRM2 expression observed in both MDA‐MB‐231 and MCF10A cells may indicate an association between the response to siRNA treatment and pathways involved in nucleotide metabolism, DNA synthesis, and cellular proliferation. The differential regulation of ASNS additionally suggests a cell type‐dependent metabolic stress response. These observations identify candidate pathways for further mechanistic investigation, although direct regulation of RRM2 or ASNS by LFG cannot be concluded from the present data.

Taken together, the results demonstrate that treatment with LFG‐specific siRNA was associated with a characteristic molecular response in MDA‐MB‐231 cells comprising reduced LFG mRNA expression, altered intracellular calcium‐sensitive fluorescence, and differential regulation of genes associated with ER stress, metabolism, and apoptosis. The combination of these observations indicates an association between LFG‐targeted siRNA treatment, reduced LFG mRNA expression, and changes in cellular pathways related to ER homeostasis in triple‐negative breast cancer cells. However, the present data do not establish that these downstream changes result directly from depletion of LFG protein. The findings are also consistent with broader pan‐cancer analyses identifying FAIM2 as a tumor‐associated and prognostically relevant molecule [19].

Several limitations should be considered when interpreting the findings. The study included one triple‐negative breast cancer cell line and one non‐tumorigenic mammary epithelial cell line, which limits the generalizability of the results. In addition, LFG regulation was demonstrated at the mRNA level, whereas depletion of LFG protein was not established in the present study. Therefore, the observed changes in calcium‐sensitive fluorescence and gene expression cannot be causally attributed to loss of LFG protein. These limitations define the scope of the conclusions but do not diminish the value of the observed transcriptional, calcium‐associated, and cell type‐dependent response patterns.

Future investigations should determine whether the observed reduction in LFG mRNA expression is accompanied by a corresponding reduction in LFG protein abundance using independent biological replicates and quantitative protein‐level analyses. Studies using additional LFG‐targeting siRNA sequences, functional rescue experiments, and direct apoptosis measurements would be valuable for establishing causality. Given the reported pan‐cancer relevance of FAIM2 [19], its epigenetic regulation through DNA methylation [20], and its association with tumor progression in additional malignancies [11, 16, 17, 21], further examination of LFG/FAIM2 in breast cancer is warranted.

In conclusion, the present study identifies a molecular response associated with LFG‐specific siRNA treatment and reduced LFG mRNA expression in MDA‐MB‐231 cells, involving changes in intracellular calcium‐sensitive fluorescence and genes associated with ER stress, metabolism, and apoptosis. Because LFG protein depletion was not established, these findings should be interpreted as associations rather than evidence of downstream effects caused by loss of LFG protein. The combined findings extend current knowledge of cellular responses associated with LFG‐targeted siRNA treatment in triple‐negative breast cancer cells and provide a rationale for further investigation of the functional and potential therapeutic relevance of LFG/FAIM2.

## Author Contributions

**Inga Nebel:** conceptualization, writing – original draft, investigation, methodology, visualization, writing – review and editing, formal analysis, data curation. **Sarah Strauß:** resources, writing – review and editing. **Frederik Schlottmann:** writing – review and editing. **Lena Maria Herrmann:** investigation. **Peter M. Vogt:** funding acquisition, writing – review and editing, supervision. **Vesna Bucan:** conceptualization, investigation, funding acquisition, methodology, validation, visualization, writing – review and editing, formal analysis, project administration, data curation, supervision, resources.

## Funding

The authors have nothing to report.

## Ethics Statement

The authors have nothing to report.

## Conflicts of Interest

The authors declare no conflicts of interest.

## Data Availability

The data that support the findings of this study are available from the corresponding author upon reasonable request.
